# Comparison of simultaneous bilateral versus unilateral total knee replacement on pain levels and functional recovery

**DOI:** 10.1186/s12891-020-03269-3

**Published:** 2020-04-15

**Authors:** Ahmad H. Alghadir, Zaheen A. Iqbal, Shahnawaz Anwer, Dilshad Anwar

**Affiliations:** 1 Rehabilitation Research Chair, College of Applied Medical Sciences, King Saud University, P.O.Box-10219, -11433, Riyadh, Saudi Arabia; 2 Department of Building and Real Estate, Hong Kong Polytechnic University, Kowloon, Hong Kong Special Administrative Region Hong Kong; 3 Bone Joint and Trauma Clinic, Darbhanga, India; 4 Royal Hospital and Trauma Center, Darbhanga, India

**Keywords:** Total knee arthroplasty, Bilateral, Unilateral, Pain, Function

## Abstract

**Background:**

Total knee replacement is a common operative procedure to improve pain, function, and quality of life in patients with end stage knee osteoarthritis. The current study aimed to compare simultaneous bilateral versus unilateral total knee replacement on pain intensity and recovery of function.

**Methods:**

A total of 80 patients (bilateral 50, unilateral 30) aged 63.28 (9.4) years undergone total knee replacement participated in the current study. The participants were admitted for 5–7 days in the hospital. Participants in both the group received similar inpatient and outpatient physiotherapy sessions. Pain intensity and function capacity were assessed at baseline, day 7, and day 30 postoperatively using visual analogue scale and lower extremity functional scale, respectively. Repeated measures analysis of variance was used to analyze the data.

**Results:**

Both groups showed a significant reduction of pain intensity (Day 0, mean 8.9, SD 1.0; Day 30, mean 2.2, SD 1.3 in bilateral total knee replacement; Day 0, mean 8.8, SD 1.1; Day 30, mean 2.0, SD 1.5 in unilateral total knee replacement; *p* < 0.001) and improvement in the functional capacity (Day 0, mean 16.2, SD 10.1; Day 30, mean 55.6, SD 14.6 in bilateral total knee replacement; Day 0, mean 19.1, SD 9.1; Day 30, mean 56.7, SD 15.8 in unilateral total knee replacement; *p* < 0.001) following total knee replacement at 30 days post-operatively. However, there was a non-significant difference noted between bilateral versus unilateral total knee replacement on the reduction of pain intensity (mean changes, 6.9 versus 6.8) and improvement in the functional capacity (mean changes, 39.4 versus 37.6) at 30 days post-operatively (*p* > 0.05).

**Conclusion:**

Simultaneous bilateral total knee replacement was associated with a similar reduction of pain intensity and recovery of function compared to unilateral total knee replacement, suggesting the use of simultaneous bilateral total knee replacement in patients with bilateral knee osteoarthritis since its costs and rehabilitation process could be reduced compared to staged bilateral total knee replacement.

## Background

Total knee replacement (TKR) is a common operative procedure to improve pain, function, and quality of life in patients with severe grade knee osteoarthritis (OA) [1–4]. However, in many patients, bilateral TKR is required due to involvement of bilateral OA or other arthritis [5]. After 10 years of primary TKR, the incidence of TKR for contralateral knee for end-stage OA is 37% [6]. Bilateral TKR could be performed simultaneously or in a staged. Simultaneous TKR is defined as the replacement of both knees in a single surgery. The major advantage of this surgery is that it requires only one hospital stay and rehabilitation period to recover both knees. However, previous studies have shown different perioperative risks between staged bilateral TKR and simultaneous bilateral TKR. While some studies indicate significantly higher mortality and morbidity risk with simultaneous bilateral TKR, other studies indicate reduced risk of mechanical malfunction and periprosthetic joint infection [7–10]. Additionally, an estimated cost of simultaneous TKR is almost half compared to staged bilateral TKR [11–13].

A few studies also investigated differences in pain and physical function following simultaneous bilateral or staged bilateral TKR. While one study indicates functional improvements following simultaneous bilateral TKR [14], another study reports positive outcome with respect to pain and physical function following staged bilateral TKR [15]. However, lack of control groups in these studies reduce the external validity of the results.

Many studies also compared perioperative outcomes and functional recovery between simultaneous bilateral versus unilateral TKR. For instance, Hart et al. [16] reported a reduced perioperative complication and was not correlated with more readmissions than unilateral TKR. Similarly, Borges et al. [17] reported no increase in complications or cost of simultaneous bilateral TKR surgery as compared to unilateral TKR surgery. Additionally, March et al. [18] compared the functional recovery and general health between simultaneous bilateral and unilateral TKR. They found better functional recovery and general health in simultaneous bilateral TKR group. However, participants in simultaneous bilateral TKR group were significantly younger than unilateral TKR group (70.9 versus 67.8 Y, *p* = 0.01). While a previous study reported significantly better postoperative functional outcomes in simultaneous bilateral TKR group [19], a recent study reported no differences in the functional recovery between simultaneous bilateral and unilateral TKR [20]. Therefore, the present study aimed to assess whether simultaneous bilateral TKR results comparable improvement in pain intensity and functional recovery than unilateral TKR.

## Methods

### Patients and procedure

It was a series of prospective TKR cases performed by an Orthopedic surgeon in 3 years (2016 to 2019). This study compared two surgical procedures (e.g., simultaneous bilateral versus unilateral TKR) on pain and physical function. Pain intensity and recovery of function was assessed at baseline, day 7, and day 30 post-operatively in patients with unilateral and simultaneous bilateral TKR. Institution ethics committee, RRC, King Saud University, Riyadh, Saudi Arabia approved the study. A written informed consent was taken from each patient. Inclusion criteria were as follow: (a) patients with end stage primary OA, (b) bilateral symptomatic knee OA, and (c) patients undergone first time for simultaneous bilateral or unilateral TKR. A total of 80 patients (bilateral, 50; unilateral, 30), undergoing TKR, were included in the current study. Patients with cardiopulmonary comorbidities and systemic illness such as chronic obstructive pulmonary disease, diabetes mellitus, cerebrovascular disease, peripheral vascular disease or active coronary artery disease were excluded for simultaneous bilateral or unilateral TKR [16]. All patients went through a preoperative medical evaluation to rule out high risk patients for simultaneous bilateral or unilateral TKR. The participants were admitted for 5–7 days in the hospital.

### Operative procedures

Medial parapatellar approach was used for both unilateral and simultaneous bilateral TKR [21]. Vanguard® knee system and the Triathlon® Knee System prostheses were used. Knee joint was opened, osteophytes were removed, and resurfacing was done. Intramedullary drilling was done into femoral canal via intercondylar notch. Intramedullary distal resection guide was placed at 6 degrees of valgus and standard 9 mm distal resection was done to match with the distal thickness of the implant. Anterior referencing guide was used to measure femoral size. A chamfer was placed, and anterior, posterior, and oblique resections were made. Proximal tibial resection was done using extramedullary referencing guide and seven degrees of posterior slope was made. Then, tibia sling and broaching was done. Trial implant was placed, and stability and patellar tracking was assessed. When it was found satisfactory, implant placed, and cementing done. Wound was cleaned using Pulsed lavage technique [22]. Finally, closure was done in layers as suggested [23].

### Pre- and post-operative physiotherapy procedures

Participants in both the group received similar inpatient (30 min, two sessions a day for 5 to 7 days as required) and outpatient (one session, 5 days a week for 3 weeks) physiotherapy sessions. Inpatient physiotherapy sessions comprised of strength training of lower extremity (e.g., hamstrings, quadriceps, and glutei muscles), mobility exercise, range of motion exercise, and gait training with walker. Outpatient physiotherapy sessions includes strength training of hamstrings, quadriceps, and glutei muscles, mobility exercise, range of motion exercise, and gait training and walking reeducation.

### Outcomes

Pain intensity and function capacity were assessed at baseline, day 7, and day 30 post-operatively using visual analogue scale (VAS) and lower extremity functional scale (LEFS), respectively. The VAS is a valid and reliable outcome measure to assess both acute and chronic pain [24–26]. VAS is a 10 cm self-reported scale connected by 0 (indicates no pain at all) and 10 (indicates maximum pain). The 20-item LEFS is a reliable and valid functional outcome to assess lower-extremity function in patients undergoing knee or hip arthroplasty [27, 28]. The LEFS is a 5-point Likert scale ranging from 0 to 4. Total possible scores range between 0 and 80 points, where a higher score indicates a better functional capacity.

### Statistical analysis

Data was analyzed using IBM SPSS Statistics 21. The improvement in pain and functional scores during 1-month between simultaneous bilateral versus unilateral TKR were assessed using the repeated measure ANOVA. Two variables for group (simultaneous bilateral versus unilateral TKR) and three variables for time (0 day versus 7 day versus 30 day) were used. A value of *p* < 0.05 was considered for the statistical significance. The sample size was calculated using G*Power version 3.1.9.4. The required sample size for detecting an effect of 0.25 with 80% power and 0.05 level of significance in comparison of two treatment group ((simultaneous bilateral versus unilateral TKR) and three level of measurements (baseline, day 7, day 30) was 86. However, in the current study, only 80 patients were included.

## Results

**Fig. 1:**
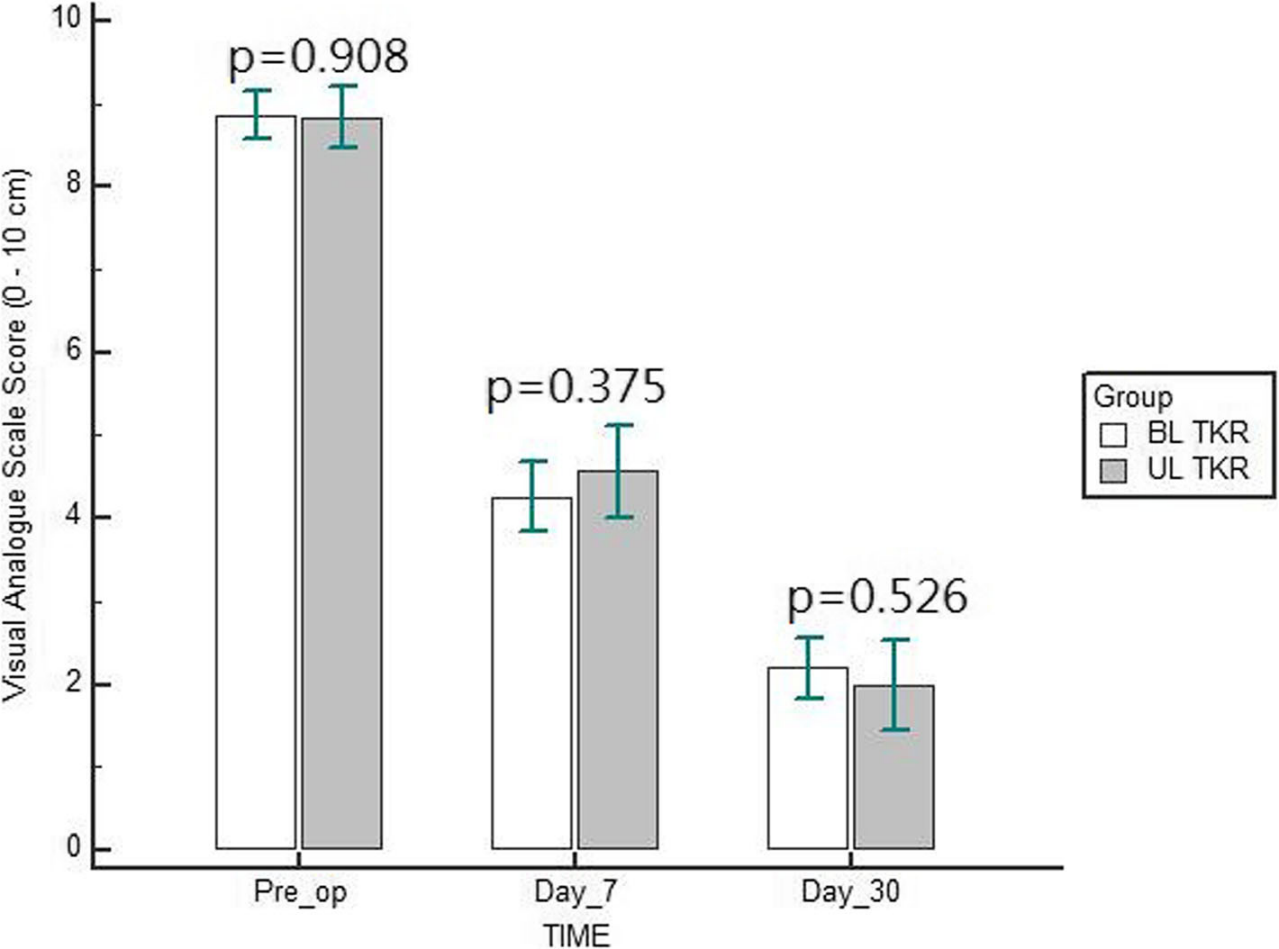
Comparison of visual analogue scale score between simultaneous bilateral and unilateral total knee replacement (TKR)

**Fig. 2:**
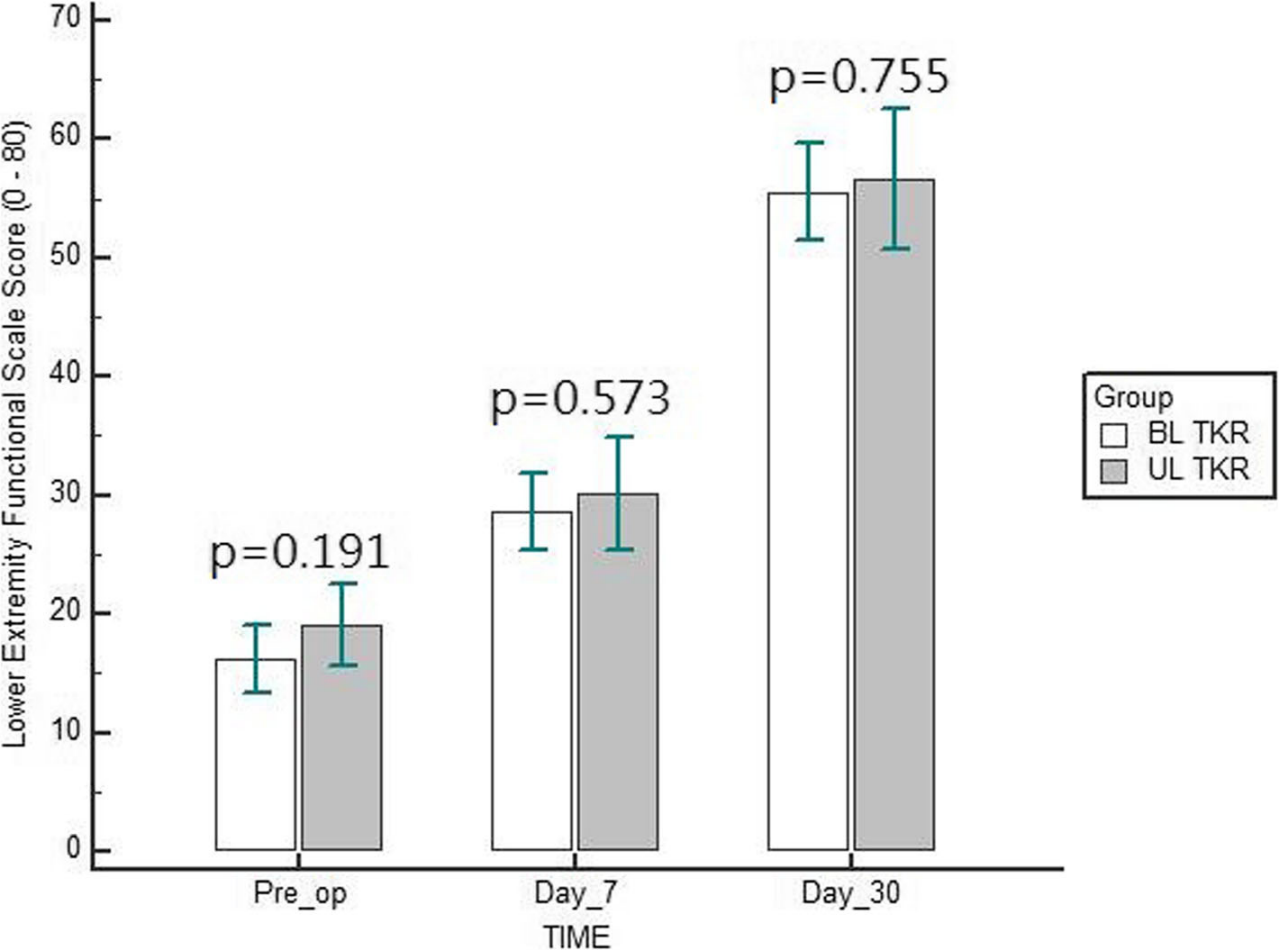
Comparison of lower extremity functional scale score between simultaneous bilateral and unilateral total knee replacement (TKR)

Table 1 details the participant’s characteristics. Mean age was 61.8 (SD, 9.2) and 65.7 (SD, 9.4) years in simultaneous bilateral TKR and unilateral TKR group, respectively. Both groups showed a significant reduction of pain intensity and improvement in the functional capacity following TKR at 30 days post-operatively (*p* < 0.001) (Table 2). However, there was a non-significant difference noted between simultaneous bilateral versus unilateral TKR on reduction of pain intensity and improvement in the functional capacity at 30 days post-operatively (*p* > 0.05) (Figs. 1 and 2).

**Table 1:** Participant’s characteristics

Demographic and clinical variables		Simultaneous bilateral TKR (*n = 50*)	Unilateral TKR (*n = 30*)	*p*-value
Age (years)		61.8 (9.2)	65.7 (9.4)	0.075
Gender	Male	18 (36%)	11 (37%)	0.952
	Female	32 (64%)	19 (63%)	
Weight (kg)		93.5 (8.2)	96.1 (5.5)	0.014
VAS	Day 0	8.9 (1.0)	8.8 (1.1)	0.908
	Day 7	4.3 (1.5)	4.6 (1.5)	0.375
	Day 30	2.2 (1.3)	2.0 (1.5)	0.526
LEFS	Day 0	16.2 (10.1)	19.1 (9.1)	0.191
	Day 7	28.6 (11.5)	30.2 (12.8)	0.573
	Day 30	55.6 (14.6)	56.7 (15.8)	0.755

*TKR* Total knee replacement; *VAS* Visual analog scale (0–10 cm); *LEFS* Lower extremity functional scale (0–80); Data are mean (Standard deviation)

**Table 2:** Comparison of VAS and LEFS scores in two groups

		Simultaneous bilateral TKR (*n = 50*)	Unilateral TKR (*n = 30*)	F	*p*-value
VAS	Day 0	8.9 (1.0)	8.8 (1.1)	0.032	0.859
	Day 7	4.3 (1.5)	4.6 (1.5)		
	Day 30	2.2 (1.3)	2.0 (1.5)		
	Change	6.7	6.8	0.65	0.522
	*P*-value (intra group)	< 0.001			
LEFS	Day 0	16.2 (10.1)	19.1 (9.1)	0.59	0.447
	Day 7	28.6 (11.5)	30.2 (12.8)		
	Day 30	55.6 (14.6)	56.7 (15.8)		
	Change	−39.4	−37.6	0.27	0.765
	*P*-value (intra group)	< 0.001			

*TKR* Total knee replacement; *VAS* Visual analog scale (0–10 cm); *LEFS* Lower extremity functional scale (0–80); Data are mean (Standard deviation)

## Discussion

The current study aimed to compare simultaneous bilateral versus unilateral TKR on pain intensity and recovery of function at 30-days postoperatively. Results of the current study indicated that both groups showed a significant pain relief and improved function after TKR at 30 days post-operatively. There was no significant difference noted between simultaneous bilateral versus unilateral TKR on pain intensity and recovery of function.

Some studies indicate that simultaneous bilateral TKR surgery reduces rehabilitation time and have no additional risk for postoperative complications compared to unilateral TKR [29–32, 32–35]. Additionally, the patient satisfaction scores, and functional outcomes are comparable, or better, in patients undergoing bilateral TKR than unilateral TKR, and this achieves without any additional medical costs [18, 29, 18, 32]. While other studies reported statistically insignificant differences in pain reduction and functional recovery between bilateral versus unilateral TKR [29, 33, 32, 36], many studies indicated an increased postoperative complications and higher rehabilitation costs, in patients undergoing bilateral TKR than unilateral TKR [9, 34, 35, 9, 37, 38].

Recently, a study reported that bilateral simultaneous unicompartmental knee arthroplasty shows better functional recovery at 6 month post-operatively than unilateral TKR [36, 39]. However, a direct comparison could not be made as many methodological differences existed between previous and current study. First, previous study compared bilateral simultaneous unicompartmental knee arthroplasty with unilateral TKR; in contrast, the current study compared bilateral simultaneous TKR with unilateral TKR. Second, previous study compared outcome at 6 months postoperatively, in contrast, the current study compared outcome at 1 month postoperatively.

TKR is most common and successful surgical intervention to reduce pain and improve function in patients with end stage osteoarthritis [37, 38, 29, 30]. There are many factors should be considered before deciding surgical intervention such as patient’s age, severity, symptom duration, pre-operative medical condition, and unilateral or bilateral involvement [31, 39]. The commonest indications for TKR include OA, traumatic arthritis and rheumatoid arthritis [31, 39]. In the current study, all patients had a diagnosis of primary knee OA.

It has been recommended that patients undergo simultaneous bilateral TKR surgery had a prolong rehabilitation, increased length of hospital stay, higher blood transfusion, increased number of painful postoperative days, a greater number of complications, and increased financial burden [31, 39]. Nonetheless, these parameters have been showed significantly better than in those patients undergo staged arthroplasty surgery [13, 18, 40]. Although several studies indicated that postoperative medical complications often seen in patients undergo simultaneous bilateral TKR surgery [41–43], other studies indicated similar complication rates [44, 45].

It is well established that TKR reduces knee pain and improves physical function in patients with knee OA. In line with previous studies, the current study reported reduced pain intensity and improved physical function in both simultaneous bilateral or unilateral TKR groups. The changes in pain intensity and physical function were statistically and clinically significant and were greater than reported minimally clinical important difference [46–48]. The current study reported a higher reduction in pain intensity in both groups than previous study (75% versus 47%) [49]. In contrast to previous study, simultaneous bilateral TKR group reported little higher functional improvement than unilateral TKR group (71% versus 66%) in the current study [49]. However, there were some methodological differences exists between current and former study. Number of simultaneous bilateral TKR group was large (63% versus 27%) in the current study while in the previous study unilateral TKR group was large (69% versus 31%). Additionally, previous study used the Western Ontario McMaster universities osteoarthritis index while the current study used LEFS to assess physical function.

The current study has several potential limitations. In the current study, physical function was assessed using LEFS, which is a subjective self-report functional scale. An objective outcome measure could be included to assess wide range of physical function. For instance, various performance based outcome measures such as timed up and go test and stair climbing test could be used to better understand functional recovery in these population. Additionally, the current study only assessed pain and function. Other important outcome measures such as ambulation, muscle strength, mobility, range of motion, and quality of life are warranted to consider in future study. The result of this study was restricted to simultaneous bilateral or unilateral TKR in patient with end stage OA, and therefore it might limit the generalizability of findings to other types of replacement surgeries. Additionally, the current study compared simultaneous bilateral TKR with a single unilateral TKR instead a staged bilateral TKR. Therefore, randomized controlled studies are warranted to further identify the differences in the various outcomes between simultaneous and staged bilateral TKR. Moreover, future study may investigate the effect of physiotherapy intervention to reduce post-operative complications and improve functional outcomes after simultaneous bilateral or unilateral TKR.

## Conclusions

Simultaneous bilateral TKR was associated with similar reduction of pain intensity and recovery of function compare to unilateral TKR, suggesting the use of simultaneous bilateral TKR in patients with bilateral knee osteoarthritis since its costs and rehabilitation process could be reduced compared to staged bilateral TKR.

## Data Availability

All data generated or analyzed during this study are presented in the manuscript. Please contact the corresponding author for access to data presented in this study.

## Abbreviations

TKR: Total knee replacement
VAS: Visual analogue scale
LEFS: Lower extremity functional scale
OA: Osteoarthritis
